# Is uromodulin a reliable biomarker in pediatric vesicoureteral reflux? An exploratory pilot study

**DOI:** 10.1007/s11255-026-05289-9

**Published:** 2026-07-20

**Authors:** Antonio Corsello, Rosa Cusumano, Ciro Corrado, Giuseppe Puccio, Maria Michela D’Alessandro, Silvio Maringhini

**Affiliations:** 1 Department of Clinical-Surgical, Diagnostic and Pediatric Sciences, University of Pavia, Pavia, Italy; 2 Children’s Hospital “G. Di Cristina”, ARNAS Civico-Di Cristina-Benfratelli, Palermo, Italy; 3 AUP “Paolo Giaccone”, Palermo, Italy; 4 Department of Pediatrics, Surgery and Transplantation, Pediatric Unit, ISMETT-IRCCS, Palermo, Italy

**Keywords:** Uromodulin, Vesicoureteral reflux, VUR, Urinary tract infection, UTI, Renal scars

## Abstract

**Purpose:**

Vesicoureteral reflux (VUR) is associated with recurrent febrile urinary tract infections (FUTIs) and renal scarring in children. Uromodulin (UMOD), one of the most abundant urinary proteins, has been proposed as a protective factor against UTIs and a marker of tubular function. Whether urinary UMOD (uUMOD) concentrations differ between children with VUR with and without renal scars remains unexplored.

**Methods:**

This was an exploratory, hypothesis-generating pilot study. We enrolled 42 children with VUR, at least one documented FUTI, normal eGFR, and no other urinary tract malformation, along with 17 age- and sex-matched healthy controls. uUMOD concentration (µg/ml) was measured in first-morning spot urine by ELISA. Children with renal scars on DMSA scintigraphy were classified as Group A (n = 22); those without scars as Group B (n = 20). The uUMOD/urinary creatinine ratio (uUMOD/uCre, expressed as mg/g) was used as the primary creatinine-corrected biomarker endpoint to adjust for urinary dilution. No formal a priori power calculation was feasible given the absence of prior pediatric data in this specific setting; sample size was determined by convenience sampling.

**Results:**

Absolute uUMOD concentrations were significantly lower in children with VUR compared to controls (p = 0.005), and lowest in Group A. However, the uUMOD/uCre ratio did not differ significantly among the three groups (Group A: 29.0 mg/g; Group B: 38.5 mg/g; controls: 36.5 mg/g; p = NS). Multivariate analysis confirmed strong collinearity between uUMOD and urinary creatinine (p<0.0001): when both were included in the regression model, only uCre retained independent significance (p = 0.01), while uUMOD did not (p = 0.18). No significant association was found between uUMOD/uCre and renal scarring, FUTI frequency, or VUR grade.

**Conclusion:**

In children with VUR, the reduction in absolute uUMOD most likely reflects impaired tubular concentrating capacity rather than an independent downregulation of uromodulin secretion. This conclusion is inferred from the strong collinearity pattern and should be confirmed in future studies including direct measures of urinary concentrating ability (osmolality, specific gravity). Spot uUMOD should not be used as a standalone biomarker for renal scar risk stratification in VUR. Future prospective studies should adopt creatinine-corrected measurements or timed urine collections as primary endpoints, with adequate sample sizes, to properly evaluate uromodulin as a clinical biomarker in this population.

## Background

Vesicoureteral reflux (VUR) is a common urinary tract abnormality in children, affecting approximately 1–2% of the pediatric population [1]. VUR is associated with recurrent urinary tract infections (UTIs) and renal scars. UTIs are among the most common bacterial infections in childhood; fever associated with UTI (FUTI) indicates kidney infection, and recurrent FUTIs may produce permanent kidney damage resulting in consequent hypertension, complications during pregnancy, and renal insufficiency [2–4]. Although the renal damage is correlated with the grade of reflux and the number of UTIs involving the kidneys, it is possible that other factors have a relevant role in producing renal scars [5–7]. The role of antibiotic prophylaxis in VUR is controversial [5–7]. VUR is traditionally diagnosed by voiding cystourethrography using water-soluble contrast or radionuclide cystography. More recently, contrast-enhanced voiding has emerged as a valid, radiation-free alternative [8]; the results of all these investigations can be affected by several factors, including operator skill. VUR is usually classified in five grades [9] and grades III–V, associated with dilatation of the urinary excretory system, are considered high grade. The current radiological investigation for diagnosing RS is ⁹⁹ᵐTc-dimercaptosuccinic acid (DMSA) scintigraphy. A DMSA kidney scan does not differentiate between congenital and acquired RS.

The identification of biomarkers that could noninvasively identify children at risk for subsequent febrile infections and the understanding of the molecular basis of the increased risk of renal scarring among children with recurrent FUTIs has been claimed as a research priority [6, 10]. Uromodulin (UMOD), also known as Tamm-Horsfall protein, represents the most common urinary protein, and it is mostly produced in the thick ascending limb cells of Henle’s loop and released into the lumen [11, 12]. It has been documented that UMOD may cause the induction of pro-inflammatory cytokine release from human monocytes, neutrophil attachment, and activation of myeloid dendritic cells [13–15]. Deposits of UMOD have been detected in human kidneys from patients with "chronic pyelonephritis", reflux nephropathy, or obstructive uropathy [16–19]. For these reasons, a role for UMOD in the development of renal scars has been suggested, though the exact mechanisms remain incompletely understood. Moreover, UMOD protects against ascending UTI by binding with type I–fimbriated Escherichia coli, preventing bacterial adhesion to the uroepithelium and facilitating bacterial clearance through urinary washout [20].

Clinical studies have shown that higher urinary uromodulin concentrations are associated with a lower risk of UTIs [21]. We hypothesize that a defect in UMOD production may increase susceptibility to UTI and predispose to renal scarring. Clinical data on uUMOD in children, and specifically in children with VUR, are virtually absent, making this an unexplored area warranting exploratory investigation. Our study aimed to evaluate uUMOD concentrations in children with VUR and to relate them to FUTIs and renal damage. It must be noted that all enrolled VUR patients had a history of at least one FUTI, which was a mandatory inclusion criterion; no VUR group without FUTI and no non-VUR FUTI group were included. This limits the ability to disentangle the individual contributions of VUR, prior FUTIs, and renal scarring to observed uUMOD differences, a limitation explicitly acknowledged throughout.

## Materials and methods

### Study design

This was an ambispective, observational, exploratory, hypothesis-generating pilot study. Clinical history variables (number of previous UTIs, VUR grade, prior surgical interventions, and DMSA scan results) were retrieved retrospectively from medical records. The primary biological outcome (uUMOD concentration) and renal function were measured prospectively in a cross-sectional manner at the time of enrollment.

### Patients

Children affected by VUR who consecutively attended the outpatient clinic of our Pediatric Nephrology Unit at Palermo Children's Hospital during a six-month period and provided informed consent were enrolled. Inclusion criteria were: age 0–20 years, VUR documented by voiding cystourethrography, at least one documented FUTI, DMSA scan performed at least six months after the last FUTI, and normal eGFR (>90 ml/min/1.73 m2). Exclusion criteria were renal insufficiency, other urinary tract malformations, neurogenic bladder, hypertension, active UTI at time of urine collection, and lack of compliance. Age- and sex-comparable children with no history of UTI or kidney disease, living in the same community, were enrolled as controls.

Clinical variables recorded included: date of birth, sex, age at first diagnosis, duration of follow-up, grade and side of VUR, family history of VUR, DMSA report, medical therapy (including antibiotic prophylaxis and breakthrough infections), number of symptomatic FUTIs (based on medical history), surgical therapy, and voiding habits. Physical measurements (height, weight, blood pressure) were performed at enrollment. Bladder and bowel dysfunction were assessed by clinical history and voiding diary where available; however, formal validated questionnaire scoring was not systematically applied, which is acknowledged as a limitation. Urine sampling was performed at least one month after resolution of the last UTI, minimizing interference from acute inflammation; the interval between last UTI and sampling was recorded for all patients.

### DMSA evaluation

Renal scars identified by DMSA were evaluated by two independent observers blinded to clinical data. Original DMSA images were re-reviewed specifically for this study (not relying solely on historical reports). Inter-observer agreement was assessed by Cohen's kappa (κ = 0.82, substantial agreement). Children with renal scars were classified as Group A (n = 22); those without scars as Group B (n = 20). We acknowledge that DMSA scintigraphy cannot reliably differentiate congenital renal dysplasia from acquired post-infectious scarring; renal ultrasound with kidney size Z-scores was available in most patients and did not reveal overt dysplastic features, though this cannot entirely exclude congenital parenchymal abnormalities.

### Biomarker measurement

Urine samples were collected in the outpatient clinic in the morning after overnight fasting, standardized for the first-morning void. Samples were stored at −20 °C; all samples were analyzed in a single batch within 6 months of collection, with no more than one freeze-thaw cycle per sample. uUMOD was measured using a validated ELISA based on a sheep anti-human uromodulin capture antibody, a mouse monoclonal anti-human uromodulin detection antibody, and a goat anti-mouse IgG HRP-conjugated secondary antibody (#172–1011, Bio-Rad). Detection used a 250 µg/L OPD substrate solution in phosphate-citrate buffer. Human uromodulin (Millipore, Billerica, MA, USA) was used for the standard curve. The ELISA sensitivity was 2.8 µg/ml; linearity 1.028. Urinary creatinine (uCre) was measured using the Beckman Coulter Synchron® System Creatinine Assay [22]. uUMOD was expressed as µg/ml; uCre as mg/dl. The uUMOD/uCre ratio (mg/g) was used as the primary creatinine-corrected endpoint.

Although direct measures of urinary concentrating capacity (osmolality, specific gravity) were not available in this cohort, a significant limitation, the parallel decrease in both uUMOD and uCre observed in VUR patients, particularly in Group A, was interpreted as a pattern consistent with generalized reduced concentrating capacity, likely due to tubulo-interstitial damage. This inference must be regarded as hypothesis-generating and should be validated in future studies with concurrent osmolality measurements.

Cut-off for recurrent FUTIs. Children with ≥3 FUTIs were classified as having recurrent FUTIs, based on the threshold frequently adopted in pediatric nephrology literature, consistent with thresholds used in major clinical guidelines and the RIVUR and IRIS trial frameworks [1, 2].

### Statistical analysis

Categorical variables are reported as frequencies and percentages. Numerical variables with approximately normal distribution (age) are reported as mean ± SD; skewed variables (uUMOD, uCre, uUMOD/uCre) are reported as median and IQR. Normality was assessed by visual inspection and the Shapiro-Wilk test. Between-group comparisons of categorical variables used chi-square or Fisher's exact test. Between-group comparisons of numerical variables used non-parametric tests (Wilcoxon for two groups; Kruskal-Wallis for three groups); correction for multiple comparisons used Dunn's post-hoc test with Bonferroni adjustment. Linear regression was used for numerical outcomes. Ordinal regression (proportional odds model) was used for categorical outcomes with multiple ordered levels; the dependent variable was group allocation (controls/Group B/Group A), treated as an ordinal variable reflecting increasing clinical burden (absence of VUR → VUR without scars → VUR with scars). This ordinal ordering was chosen as a pragmatic approximation to capture a gradient of renal involvement, while acknowledging that this assumption is imperfect in a cross-sectional design. Confidence intervals (95% CI) are reported for all regression estimates. Effect sizes (rank-biserial correlation for non-parametric comparisons) are provided. All analyses were performed in R (R Core Team, 2025). This is an exploratory pilot study; formal a priori power calculation was not feasible. All findings, particularly subgroup analyses, must be regarded as preliminary and hypothesis-generating.

This study was approved by the ARNAS Civico-Di Cristina-Benfratelli Ethical Committee (approval number: 001186), Palermo, Italy, and was carried out in accordance with the Declaration of Helsinki.

## Results

A total of 42 patients with VUR and 17 healthy controls were enrolled. Table 1 presents the study population organised in three columns for direct comparison.

**Table 1:** Demographic and clinical characteristics of the study population and uUMOD values

Variable	Group A (VUR + Scars, n = 22)	Group B (VUR, no Scars, n = 20)	Controls (n = 17)
Age, mean ± SD (years)	8.5 ± 5.1	6.8 ± 3.9	8.1 ± 3.9
Age, median (years)	8.6	7.1	7.3
Sex (M/F)	11/11	7/13	9/8
VUR grade, mean ± SD	3.8 ± 0.9	3.5 ± 1.1	—
Bilateral VUR, n (%)	10 (45%)	14 (70%)	—
FUTIs per patient, mean ± SD	3.3 ± 1.5	2.0 ± 0.9	—
Recurrent FUTIs (≥3), n (%)	8 (36%)	6 (30%)	—
Antibiotic prophylaxis, n (%)	18 (82%)	20 (100%)	—
Breakthrough UTIs, n	3	2	—
Surgical correction, n (%)	8 (36%)	1 (5%)	—
uUMOD (µg/ml), median (IQR)	20.2 (20.3)	28.2 (18.8)	43.3 (32.5)
uUMOD/uCre (mg/g), median	29.0	38.5	36.5

*VUR* vesicoureteral reflux; *FUTI* febrile urinary tract infection; *M* male; *F* female; *uUMOD* urinary uromodulin; *uCre* urinary creatinine

By study design, all 42 VUR patients had at least one documented FUTI. Of these, 14 children (33%) had recurrent FUTIs (≥3 episodes), while 28 (67%) had ≤3. Renal scars were detected in Group A (n = 22): 11 males (mean age 6.8 ± 4.6 years) and 11 females (mean age 10.2 ± 5.1 years). Reflux grade was comparable between sexes in Group A (mean 3.8 ± 0.9), with bilateral involvement in 10 children (45%). Mean FUTIs: 3.3 ± 1.5 per patient; 18 children (82%) received continuous antibiotic prophylaxis; 8 (36%) underwent surgical correction (ureteral reimplantation). In Group B (n = 20): 7 males, 13 females; mean VUR grade 3.5 ± 1.1; bilateral in 14 (70%); mean FUTIs 2.0 ± 0.9; all on antibiotic prophylaxis; 1 surgical intervention. Age was comparable across the three groups. No significant correlation was found between renal scars and FUTI frequency (p = NS) or VUR grade (p = NS).

**Fig. 1:**
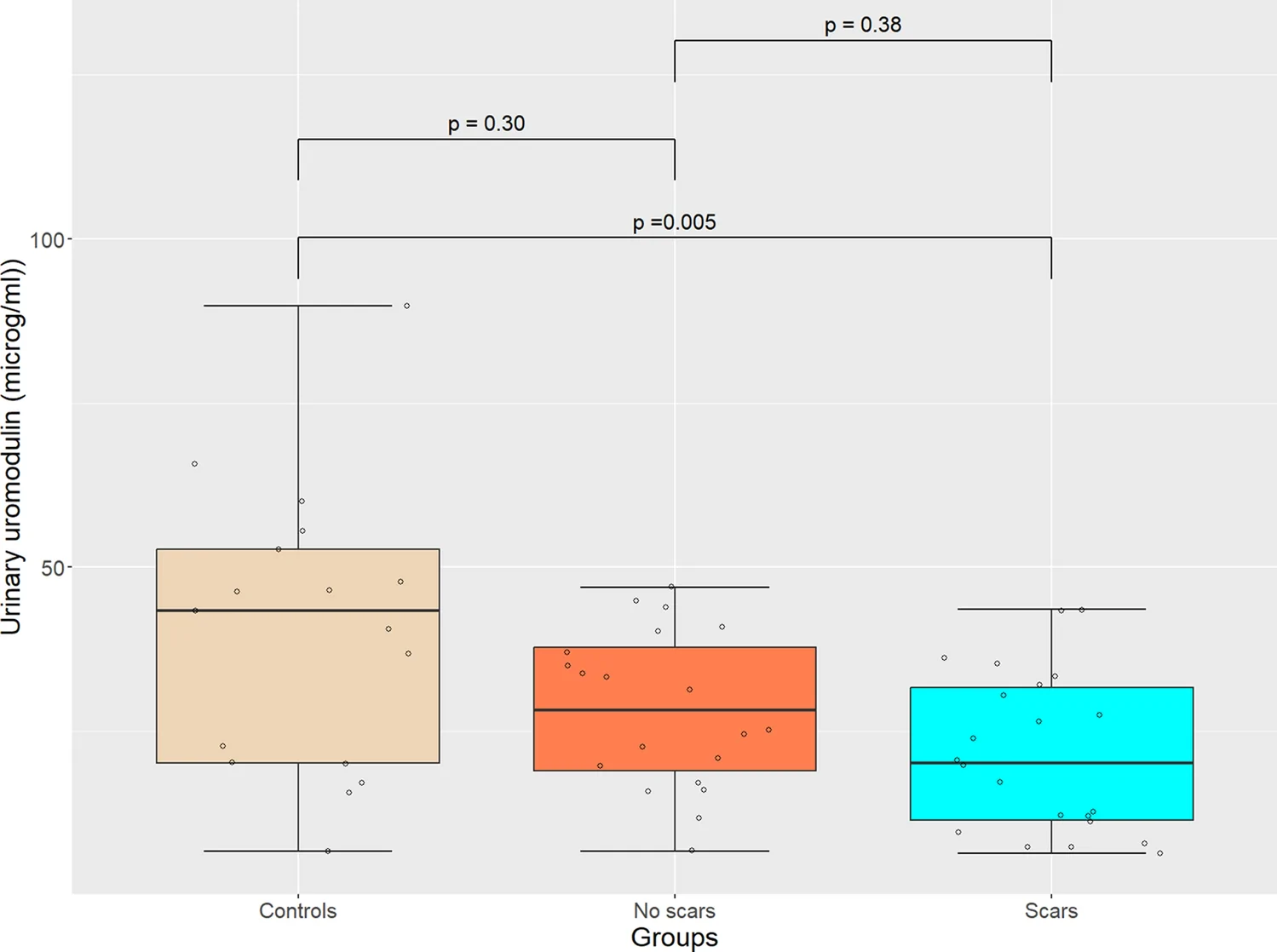
Urinary uromodulin in the three groups. p-values shown are adjusted from a Kruskal-Wallis post hoc test

Absolute uUMOD was significantly lower in children with VUR vs controls (p = 0.005; rank-biserial correlation r = −0.46, 95% CI −0.67 to −0.19) and significantly lower in Group A vs controls (p<0.05), but not significantly different between Group A and Group B (p = NS) (Fig. 1). uUMOD was numerically lower in patients with frequent (≥3) FUTIs (p = 0.07, non-significant) and comparable across VUR grades (p = 0.40).

**Fig. 2:**
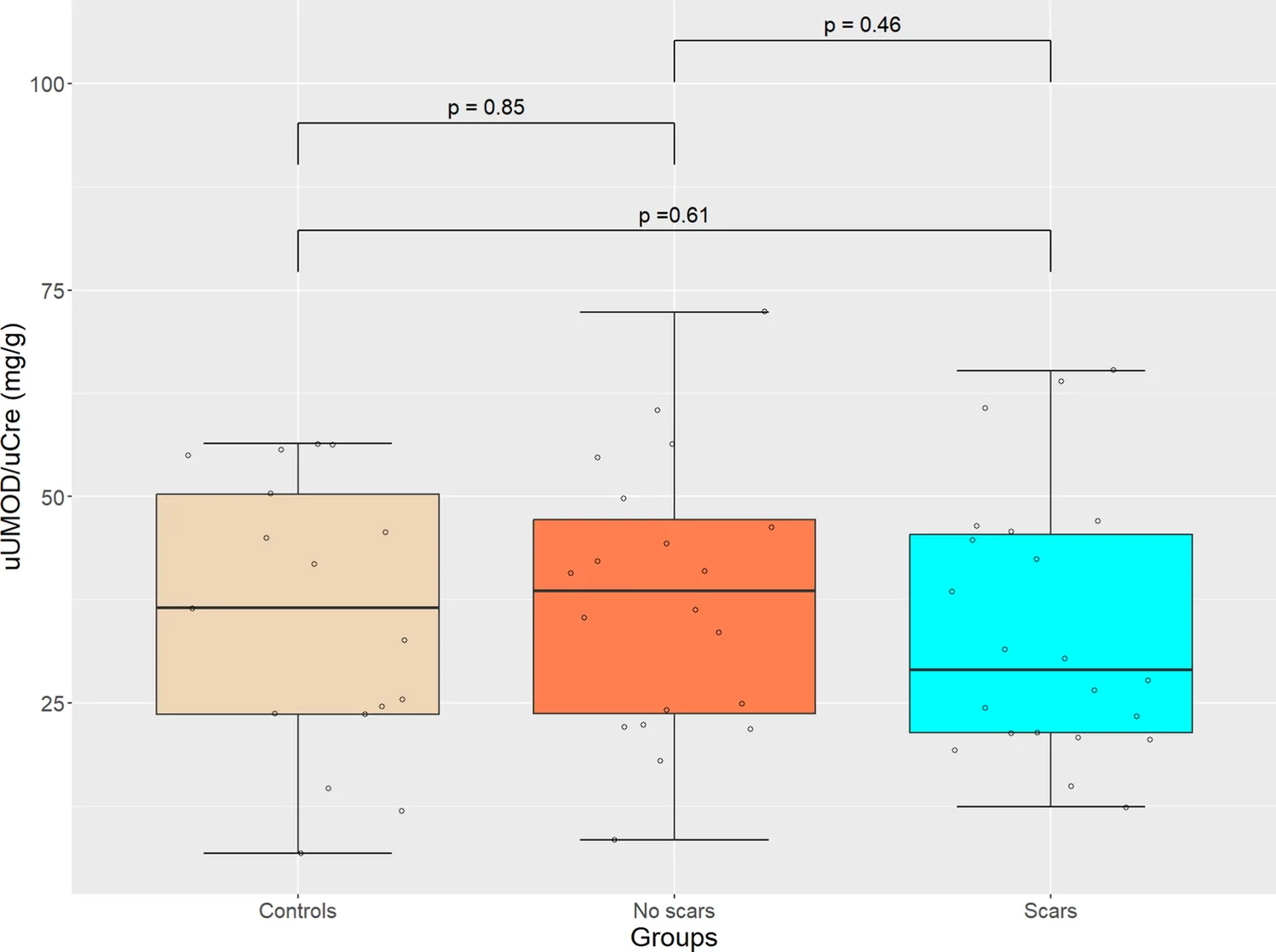
Urinary uromodulin (uUMOD)/urinary creatinine (uCre) ratio in the three groups. p-values shown are adjusted from a Kruskal-Wallis post hoc test

uUMOD/uCre ratio did not differ significantly among the three groups (Group A 29.0 mg/g; Group B 38.5 mg/g; controls 36.5 mg/g; p = NS) (Fig. 2). No significant difference was observed by FUTI frequency (25.3 vs 38.3 mg/g, p = NS) or VUR grade (32.5 vs 43.2 mg/g, p = NS).

**Fig. 3:**
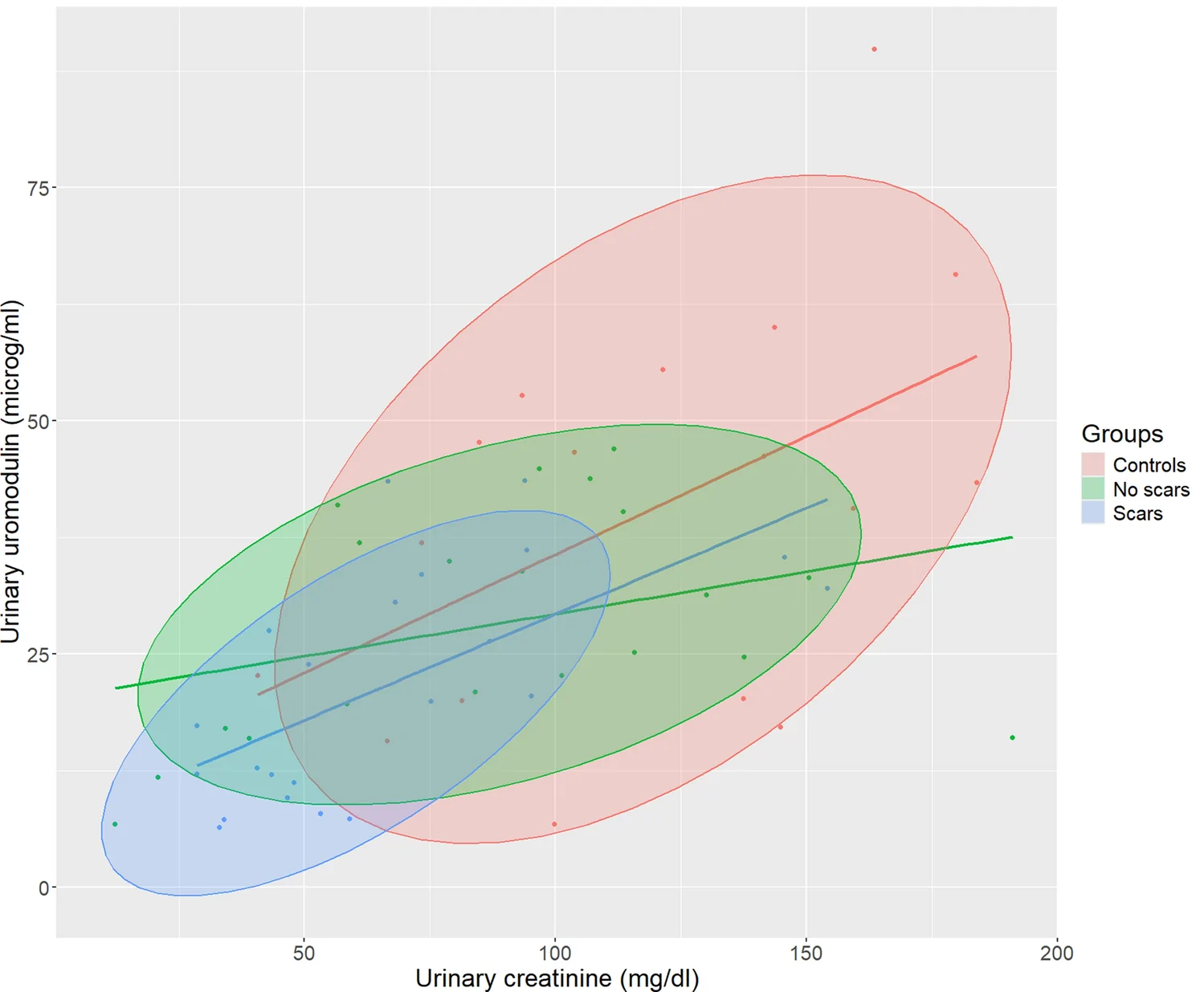
Urinary creatinine and urinary uromodulin in the three groups. The two variables are strongly collinear (p < 0.0001), and lower values of both variables are observed in the two test groups vs controls, especially in the scars group

Urinary creatinine showed a pattern of decrease across groups identical to uUMOD, with strong collinearity between the two variables (p<0.0001) (Fig. 3). Figure 3 represents one of the central findings of this study: the near-identical parallel distribution of uUMOD and uCre across groups demonstrates that lower absolute uromodulin in VUR patients reflects a generalised urine dilution pattern rather than selective uromodulin suppression. In ordinal regression with uUMOD and uCre entered separately, both were significant predictors of group allocation (p<0.05 each). When entered together, only uCre retained significance (p = 0.01, 95% CI reported; uUMOD p = 0.18), with R^2^=0.341, the main effect driven by the difference between controls and both VUR groups.

## Discussion

The principal finding of this exploratory pilot study is that the uUMOD/uCre ratio, which corrects for urinary dilution, does not differ significantly between children with VUR and renal scars, those without scars, and healthy controls. While absolute uUMOD concentrations were lower in VUR patients, particularly in Group A, this difference was fully explained by strong collinearity with urinary creatinine, a pattern consistent with reduced urinary concentrating capacity rather than a primary suppression of uromodulin secretion. This conclusion must, however, be regarded as inferential, as direct measurement of urine concentrating ability (osmolality, specific gravity) was not performed in this cohort.

Timed urine collection is the most accurate method for assessing urinary excretion of substances; since 24-hour urine collection in children is generally unreliable, spot urine biomarkers are typically corrected using the ratio with urinary creatinine, which approximates constant daily excretion [23, 24]. This method is the established standard for urinary protein and biomarker quantification in pediatric populations. However, urinary creatinine in children carries substantial inter-individual variability depending on age, sex, muscle mass, hydration status, and nutritional state [25, 26]. In the present study, uCre decreased in parallel with uUMOD across groups, which is the expected pattern if generalized concentrating capacity is impaired, but the same pattern could also reflect differences in hydration at the time of sampling, nutritional differences, or age-related creatinine excretion differences between groups. These confounders were not fully controlled, which limits the strength of any mechanistic interpretation. The age-dependence of uUMOD/uCre is particularly relevant: in the CKiD cohort of 677 children with CKD, older age was independently associated with 12–13% lower uUMOD/uCre per year (p<0.001), independent of eGFR [27]. Age-specific reference ranges for uUMOD do not yet exist in healthy pediatric populations or in children with VUR, which must be regarded as an important warning when interpreting between-group comparison.

The original study design pre-specified absolute uUMOD concentration as the primary outcome. This was methodologically suboptimal, as creatinine correction is standard practice in urinary biomarker research and should have been the pre-specified primary endpoint from the outset. The use of first-morning urine introduces an additional source of confounding that deserves specific consideration: first-morning samples are intrinsically influenced by overnight concentrating ability. If renal scarring and tubulo-interstitial injury impair overnight urine concentration, both uCre and uUMOD will be reduced in first-morning samples, introducing a confounding mechanism circular to the proposed biological hypothesis. This is a fundamental methodological limitation of the present study design.

In children with VUR, tubular concentrating defects are a recognized consequence of renal parenchymal damage. A long-term follow-up study of 77 children with VUR demonstrated that concentrating defects (reduced maximum urine osmolality) were observed in 19.5% of patients and correlated directly with GFR (r = 0.6, p<0.001); patients with bilateral DMSA scars showed significantly lower uOsm and GFR compared to those with normal DMSA, and the observed tubular function deterioration was more related to loss of renal parenchyma than to the initial VUR grade. This is highly relevant background for interpreting our findings: if scarring and tubulo-interstitial damage reduce concentrating capacity, and thereby reduce both uUMOD and uCre proportionally, the uUMOD/uCre ratio may remain stable even in the presence of significant parenchymal damage. This would explain why the ratio did not differ between groups in our study, and why it may not serve as a specific biomarker of renal scarring severity. We suggest that lower uUMOD reflects reduced tubular mass or function in most clinical settings.

Studies on uUMOD in children remain scarce. Akioka et al. examined pediatric transplant recipients with interstitial non-specific UMOD deposits in renal allografts; 80% of 10 patients had VUR into the graft, and almost half had asymptomatic bacteriuria [28]. While the pathological substrate differs fundamentally from our cohort (interstitial UMOD deposition versus urinary UMOD secretion), both observations converge in suggesting that VUR is associated with UMOD-related tubulo-interstitial changes. The genetic dimension is also relevant: variants in the UMOD promoter region (rs4293393, rs12934455, rs13335818) influence urinary and serum uromodulin levels at the population level; the C allele at rs4293393 has been associated with higher UTI susceptibility and as shown in our earlier genetic study, potentially higher risk of renal scarring in children with VUR [29]. Our current study did not include genetic analyses, an important limitation that prevents exploration of genotype-phenotype relationships in uUMOD levels.

In the broader context of urinary biomarkers for renal scarring in VUR, it is instructive to compare uromodulin with established candidates. In the most recent systematic review on this topic, including nine studies, urinary NGAL/uCre demonstrated the best diagnostic performance (sensitivity 72–84%, specificity 60–81%, AUC up to 0.86), followed by IL-8/uCre (sensitivity 85% for scarring, AUC 0.67) and serum ST2 (sensitivity 92.9%, AUC 0.79) [30]. Urinary KIM-1/uCre showed a positive correlation with scarring severity in children with VUR [31]. Importantly, all these studies applied creatinine correction as a methodological requirement, reinforcing the present finding that uncorrected absolute biomarker concentrations are unreliable in pediatric VUR populations. The key difference between uromodulin and injury markers such as NGAL or KIM–1 is biological: NGAL and KIM–1 are acutely upregulated in tubular injury, whereas UMOD reflects constitutive tubular secretory capacity. If tubular damage reduces both UMOD production and concentrating ability proportionally, the uUMOD/uCre ratio may remain stable even when absolute UMOD is reduced, which is consistent with our findings and distinguishes uromodulin from classical tubular injury biomarkers.

Several explanations should be considered on why no association was found between uUMOD/uCre and scarring, FUTI frequency, or VUR grade: (a) the small sample size, particularly after subgroup stratification, makes the study insufficiently powered to detect modest associations; (b) uCre is an imperfect dilution corrector in children, and residual confounding by age, sex, and muscle mass may mask true biological differences; (c) UMOD may not be an independent biomarker of scarring severity once the shared effect of reduced concentrating capacity on both variables is accounted for; (d) the cross-sectional design cannot capture temporal relationships, the hypothesis that low UMOD predisposes to UTI and thus to scarring requires a prospective longitudinal design; and (e) the inability to distinguish congenital from acquired lesions by DMSA may introduce heterogeneity within Group A that blurs any putative association.

In this exploratory pilot study, the uUMOD/uCre ratio did not differ significantly between children with VUR and renal scarring, those without scarring, and healthy controls. Lower absolute uUMOD concentrations in VUR patients were strongly collinear with urinary creatinine, suggesting that impaired tubular concentrating capacity, likely a consequence of tubulo-interstitial damage and renal parenchymal loss, drives the reduction in spot uUMOD rather than a primary downregulation of uromodulin secretion. This interpretation remains inferential without direct osmolality measurements. Spot uUMOD should not be used as a standalone biomarker for renal scar risk stratification in pediatric VUR. The main contribution of this study is methodological: it highlights the critical confounding effect of urinary dilution in pediatric uromodulin studies and the necessity of pre-specifying creatinine-corrected endpoints or timed urine collections from the outset. Future adequately powered, prospective, longitudinal, multi-center studies, incorporating direct measures of concentrating capacity, standardized urine collection protocols, age-specific reference ranges, genetic analyses, and serum uromodulin, are needed to determine whether uromodulin has clinical utility as a biomarker in pediatric VUR.

## Data Availability

All data are available from the corresponding author upon reasonable request.
